# Antioxidant and antimicrobial activities of ethanol and aqueous extracts from *Urtica urens*

**DOI:** 10.1080/13880209.2016.1275025

**Published:** 2017-01-13

**Authors:** Massara Mzid, Sameh Ben Khedir, Maryem Ben Salem, Wafa Regaieg, Tarek Rebai

**Affiliations:** a Laboratory of Histology Embryology and Reproductive Biology, Faculty of Medicine of Sfax, University of Sfax, Sfax, Tunisia;; b Laboratory of Pharmacology, Faculty of Medicine of Sfax, University of Sfax, Sfax, Tunisia

**Keywords:** Flavonoids, MIC, Polyphenols

## Abstract

**Context:** *Urtica urens* L. (Urticaceae) is an important and commonly used plant for its medicinal and pharmacological properties.

**Objective:** We analyzed the antioxidant and antimicrobial activities of the leaves of *Urtica urens* in ethanol (EtOH) and water (WA) solvents, employing standard analytical methods.

**Materials and methods:** Polyphenol, flavonoid and tannin content of *Urtica urens* leaves were determined, after their extraction, using EtOH (70%) and WA extracts as well as the antioxidant (DPPH, ABTS, β-carotene and FRAP) and the antibacterial (via the method of dilution tests) activities of EtOH and WA extracts.

**Results:** The 70% EtOH of *Urtica urens* showed the highest values of total phenolic (31.41 mg GAE/g DW), flavonoids (6.81 mg quercetin/g DW), tannin (8.29 mg GAE/g DW) and TEAC (560 mmol Trolox/g DW), compared to the WA. The results of DPPH for EtOH (95.56%) were higher than that of WA (64.56%) at a concentration of 40 mg/L. The extracts displayed a FRAP 106.23 for EtOH and 30.55 μmol Fe(II)/g DW for WA. The results clearly indicated that EtOH was the strongest radical scavenger (IC_50 _=_ _245.65 ± 10.2 μg/mL). Ethanol was the most effective with minimum inhibitory concentration (MIC) < 250 μg/mL. WA has no antibacterial activity.

**Discussion and conclusion:** The results indicate that leaves of *Urtica urens* could be used as natural antioxidant and antimicrobial agents.

## Introduction

The world is presently over-dependent on a few plant species (Correa et al. 2013). Diversification of production and consumption habits to include a broader range of plant species, particularly those currently identified as under-utilized, could significantly contribute to improve health and nutrition, livelihoods and ecological sustainability. Wild plants have played a significant role in supplementing staple foods by supplying trace elements, vitamins, and minerals in order to obtain a balanced diet, and they may do so again in the future. Their interest as a source of nutraceuticals has been highlighted in recent studies (Leonti 2012). Several epidemiological studies suggest that a high intake of foods rich in natural antioxidants reduces the risk of some cancers, heart, and degenerative diseases. *Urtica urens* L. (Urticaceae) leaves, have a relatively high level of protein (66%), which is of better quality if compared with the proteins of other leafy vegetables (Hughes et al. 1980). The leaves of nettle are good sources of different significant minerals and vitamins (Adamski & Bieganska 1980; Kukric et al. 2012). Nettles contain flavonoids, fatty acids, terpenes, protein, vitamins, and minerals. Stinging nettle leaves are rich in vitamin C, B groups vitamins, vitamin K, and some minerals mainly calcium, iron, magnesium, phosphorus, potassium, and sodium (Upton 2013). Nettle leaves contain nine carotenoids: Lutein, lutein isomers and β-carotene are the basic carotenoids (Guil-Guerrero et al. 2003).

*Urtica urens* is widely distributed in Tunisia and traditionally used as an herbal medicine for a wide variety of purposes. Other species of *Urtica* has been studied the microbial and antioxidant activities (Gulcin et al. 2004; Kukric et al. 2012; Sidaoui et al. 2015). But, in our knowledge, the antimicrobial and antioxidant activities of the species of *Urtica urens* from Tunisia have not yet studied.

The use of such antioxidant and antimicrobial compounds from natural sources has been considerable interest not only for the preservation of foods and improving the shelf life of food products, but also for increasing the stability of fats and oils and to control the human and plant diseases of microbial origin (Benkeblia 2004).

This study evaluates the total phenolic compounds, total flavonoid compounds, total tannin contents, vitamins (D, E and C), the antioxidant properties (DPPH, ABTS, β-carotene and FRAP), and the *in vitro* antimicrobial activities of ethanol and aqueous extracts of leaves of *Urtica urens* against strains of bacteria which known as multi-resistant organisms or involved in diverse pathology.

## Materials and methods

### Chemicals

1,1-Diphenyl-2-picrylhydrazyl (DPPH), butylated hydroxytoluene (BHT), α-tocopherol, β-carotene, and linoleic acid were purchased from Sigma Chemical Co. (St. Louis, MO). All other chemicals, namely l-ascorbic acid, Folin-Ciocalteu reagent (mixture of phosphomolybdate and phosphotungstate, Sigma) and other solvents were of analytical grade. All solutions were freshly prepared in distilled water.

### Plant material

Aerial parts of *Urtica urens* were collected from Sfax, Tunisia, during December 2014 and identified by Professor Mohamed Chaieb from the Faculty of Sciences of Sfax (Laboratory of Biology and Vegetable Ecophysiology, Faculty of Science, Sfax, Tunisia). The voucher sample was deposited at The National Botanical Research Institute Tunisia (INRAT). It was washed with distilled water and then dried at room temperature for two days, afterwards, crushed, milled in a knife mill to obtain 100 g of *Urtica urens* powder and subsequently stored in glass bottles at room temperature.

### Preparation of plant extract

The dried powder (50 g) was extracted (by a maceration method) by adding ethanol (70%) at 37 °C for 72 h. The extract was filtered through Whatman No.1 filter paper in a Buchner funnel. The filtrate was evaporated to dryness under reduced pressure in a rotatory vacuum evaporator (EYELA N1000, Tokyo, Japan) (Nassiri-Asl et al. 2009).

The other dried powder (50 g) was subjected to an extraction with water for 20 min by infusion (1/10, plant/solvent). After the treatment, the aqueous filtrate was concentrated by rotatory evaporator in order to obtain the aqueous extract of *Urtica urens* in powder form.

The dried sample of each extract was weighed and the yield of soluble constituents was determined. The dried extracts were kept in dark at +4 °C until further analyses. The yield of the extract under study was calculated by the following equation:

Yield (%)=(A1×100)/A2

Where A1 corresponds to the *Urtica urens* concentrated by evaporator and A2 corresponds to the powdered dried plant material used for extraction with ethanol or water.

### Total phenolic compound

The total phenolic compound (TPC) of extracts was determined by the Folin-Ciocalteu method (Singleton & Rossi 1965). TPC was expressed as mg gallic acid equivalents (GAE) per gram extract. Values presented are the average of three measurements.

$$\left(y=0.121\times +0.011,r^{2}=0.9819\right).$$

### Total flavonoids compound

The total flavonoid content of *Urtica urens* extracts was determined by the method described by Zhishen et al. (1999). Flavonoid content was expressed as mg quercetin equivalent (QE)/g dried extract. Values presented are the average of three measurements.

$$\left(y=0.0182x-0.004,r^{2}=0.9949\right).$$

### Determination of hydrolyzable tannin

The hydrolyzable tannin content was determined from AECS using the potassium iodide test (Saad et al. 2012). The analysis was performed in triplicate and the results were expressed in mg equivalent of gallic acid per gram of dried extract (mg GAE/g DW) using the calibration curve of gallic acid (y = 0.121x + 0.011, r^2 ^=^ ^0.9819).

### Antioxidant activity

#### DPPH assay

The DPPH radical-scavenging activity of *Urtica urens* extracts was determined using the method described by Kirby and Schmidt (1997) with some modifications. The DPPH scavenging percentage was calculated as follows:

% DPPH scavenging=[(absorbance of negative control-absorbance of sample)/absorbance of negative control]×100.

Tests were carried out in triplicate. The free radical scavenging activity of each extract was expressed in terms of Trolox equivalents (TE) (mg TE/g extract) and at the extract concentration where 50% of DPPH^•^ was attenuated (IC_50_).

#### Antioxidant capacity by ABTS^•+^ method

The 2,2-azinobis-3-ethylbenzothiazoline-6-sulfonic acid free radical (ABTS^•+^) neutralization was determined using a spectrophotometric, 96-well microplate method described by Re et al. (1999) with minor modifications. The antioxidant capacity of each extract was expressed quantitatively in terms of trolox equivalents (TE) (mg TE/g extract) and at the extract concentration where 50% of ABTS^•+^ was neutralized (IC_50_).

#### β-Carotene bleaching by linoleic acid assay

The ability of *Urtica urens* extract to prevent bleaching of β-carotene was assessed as described by Koleva et al. (2002).

#### Ferric reducing antioxidant power (FRAP assay)

A modified method of Benzie and Strain (1999) was adopted for the FRAP assay. Results are expressed in μM Fe (II)/g dry mass (DW).

### Estimation of vitamin D

Vitamin D levels was measured by colorimetric methods using commercial reagent kits (Ref: 20151 and 20091), respectively, purchased from Biomaghreb (Ariana, Tunis, Tunisia).

### Estimation of vitamin E

The extraction of vitamin E from the method has been described by both Katsanidis and Addis (1999).

### Estimation of vitamin C

Ascorbic acid was determined as described by Jacques-Silva et al. (2001).

### Antimicrobial activity

#### Microbial strains

Antimicrobial activities of *Urtica urens* extracts were tested against eight strains of bacteria: *Staphylococcus aureus* (ATCC 6538), *Pseudomonas aeruginosa* (ATCC 27893), *Bacillus subtilis* (JN 934392), *Salmonella enteritidis*, *Escherichia coli* (ATCC 25922), *Staphylococcus epidermidis* (MTCC 3615), *Micrococcus luteus* (ATCC 4698) and *Enterococcus faecalis* (ATCC 29212). These strains were kindly provided by the Centre of Biotechnology of Sfax, Tunisia.

#### Agar diffusion method

Antimicrobial activity was performed according to the method described by Vanden Berghe and Vlietinck (1991).

#### Determination of the minimal inhibitory concentration (MIC)

MIC values, which represent the lowest plant extracts concentration that completely inhibits the growth of microorganisms, were determined based on a micro-well dilution method (1998).

#### Determination of minimum bactericidal concentration (MBC)

To determine the minimum bactericidal concentration (MBC), a loop full from those tubes, which did not show any visible growth in MIC assay, was cultured on MHA and incubated at 37 °C for 18 to 24 h. The highest dilution that did not yield colony formation on MHA was considered as MBC (Testai et al. 2002).

### Statistical analysis

Values were expressed as mean ± standard error of the mean (SEM) of many parallel measurements. Differences at *p* ≤ 0.05 were considered statistically significant. Database management and statistical analysis were performed using SPSS (Microsoft Corporation) statistical software package (SPSS Inc, Chicago, IL).

## Results

### Extraction yield and polyphenol, flavonoid and hydrolyzable tannin contents

The yield of extractable components relative to the weight of dried plant material ranged from 4.768% for EtOH extract and 0.87% for WA extract. The results of the phytochemical analysis evinced the quantitative values of EtOH extract: 31.41 ± 0.31 mg GAE/g E to phenolic compound, 6.81 ± 1.72 mg R/g E to flavonoids and 8.29 ± 0.3 mg GAE/g E to hydrolyzable tannin (Table 1). For WA extract; 5.34 ± 0.21 mg GAE/g E to total phenolic compound, 29.56 ± 1.56 mg R/g E to flavonoids and 4.05 ± 0.52 mg GAE/g E to hydrolyzable tannin (Table 1).

**Table 1.** Extract yield and total flavonoids, phenolic and tannin contents from *Urtica urens* extracts.

Sample	Yield (%)	Flavonoids (mg quercetin/g DW)	Phenolics (mg GAE/g DW)	Tannins (mg GAE/g DW)
Ethanol	4.768	6.81 ± 1.72	31.41 ± 0.31	8.29 ± 0.3
Aqueous	0.87	5.34 ± 0.21*	29.56 ± 1.56*	4.05 ± 0.52*

Values are expressed as mean ± SEM of three independent determinations. *p < 0.05.

DW: dry weight.

### Antioxidant capacity by DPPH^•^ method

**Figure 1.**
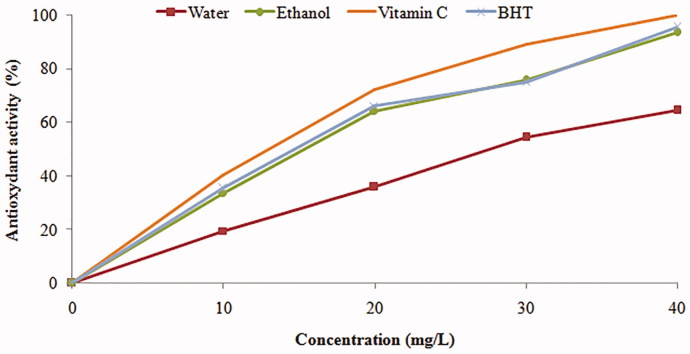
Antioxidant capacity of ethanol and aqueous extracts of *Urtica urens* by the DPPH• method at different concentrations. BHT and ascorbic acid (Vitamin C) were used as positive control. Values are means ± SEM (*n* = 3).

The DPPH method has been widely applied for estimating antioxidant activity in recent years. DPPH, a stable free radical with a purple colour, changes into a stable yellow compound upon reacting with an antioxidant. In brief, the reduction capacity of DPPH was determined by the decrease in its absorbance at 517 nm, which is reduced by the antioxidant (Duh 1998). As can be seen in Figure 1, the results clearly indicated that ethanol extract, which contained the highest amount of phenolics and flavonoids compounds, was the strongest radical scavenger (IC_50 _=_ _245.65 ± 10.2 μg/mL). The WA extract displayed free radical scavenging activity (IC_50 _=_ _142.94 ± 10.54 μg/mL) (Table 2).

**Table 2.** Antioxidant activity of *Urtica urens* extracts (ABTS^•+^, DPPH^•^).

Sample	DPPH^•^ scavenging (IC_50_, μg/mL)	ABTS^•+^ scavenging (IC_50_, μg/mL)	DPPH^•^ scavenging (mg TE/g extract)	ABTS^•+^ scavenging (mg TE/g extract)
Ethanol	245.65 ± 10.2	30.88 ± 3.03	65.33 ± 10.72	560.33 ± 29.45
Aqueous	142.94 ± 10.54***	14.65 ± 1.09***	45.67 ± 10.21***	350.33 ± 18.73***

Values are expressed as mean ± SEM of three independent determinations. ***p < 0.001.

TE: Trolox equivalents; IC: Inhibition Concentration.

The highest inhibition effect of WA and EtOH extracts of *Urtica urens* were 64.56 and 93.56%, respectively, compared with ascorbic acid with 99.83% and BHT with 95.56% at concentration of 40 mg/L. However, in our results further increase in the concentration of the *Urtica urens* extracts indicated increasing inhibition of DPPH radical (Figure 1).

### Antioxidant capacity by the method of ABTS^•+^

**Figure 2.**
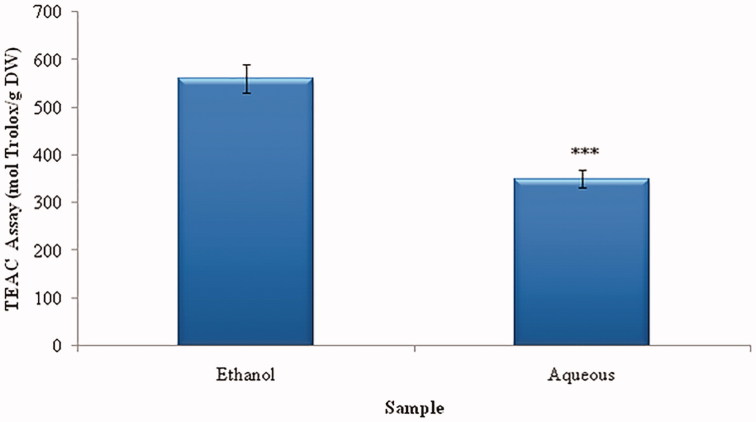
ABTS (TEAC) activities of *Urtica urens* extracts. Values are means ± SEM (*n* = 3). ***p < 0.001.

The free radical scavenging ability of *Urtica urens* phenolics was determined using ABTS radical cation, too. ABTS radical cation has often been used in the evaluation of antioxidant activity of single compounds and complex mixtures of various origins (body fluids, foods, beverages, plant extracts). In this assay, ABTS radical cation was directly generated in stable form using potassium persulfate. Generation of radicals before the antioxidants are added to prevent the interference of compounds, which affect radical formation. This modification makes the assay less susceptible to artifacts and inhibits overestimation of antioxidant capacity. EtOH extract had an activity of 560.33 ± 29.45 mol Trolox/g DW versus WA extract had much less activity compared to EtOH, 350.33 ± 18.73 mol Trolox/g DW (Figure 2).

### β-carotene bleaching by linoleic acid assay

**Figure 3.**
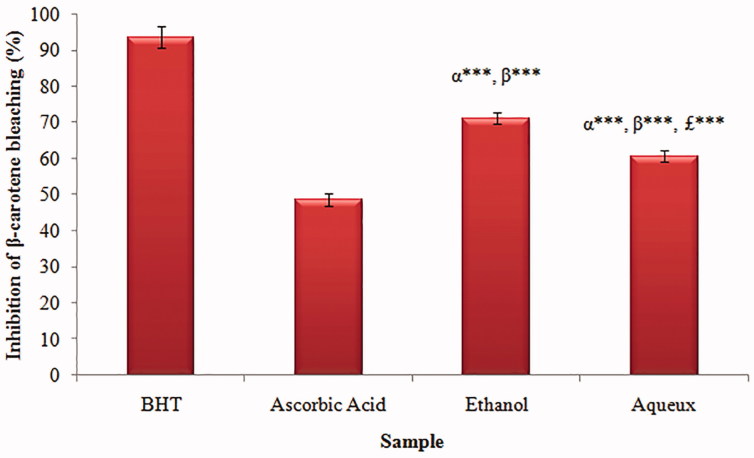
β-Carotene bleaching percentage of *Urtica urens* extracts. Values are means ± SEM (*n* = 3). ***p < 0.001. α: compared to BHT (control); β: compared to Ascorbic Acid; £: compared to Ethanol.

The β-carotene scavenging activity of the two extracts at different concentrations was compared with that of butylated hydroxytoluene (BHT), as shown in Figure 3. In this study, the addition of *Urtica urens* extracts and BHT prevented bleaching of the β-carotene at different degrees. The WA and EtOH extracts of *Urtica urens* demonstrated high ability to prevent bleaching of the β-carotene. Interestingly, they exhibited a strong inhibition of the β-carotene bleaching up to 71.12% and 60.52%, respectively, at concentrations up to 500 μg/mL.

### Ferric reducing antioxidant power ability

**Figure 4.**
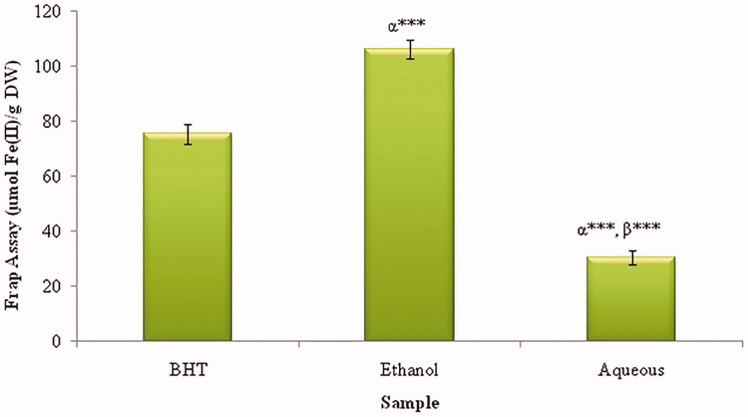
FRAP activities in ethanol and water extracts of *Urtica urens*. Values represent means ± SEM (*n* = 3). ***p < 0.001. α: compared to BHT; β: compared to Ethanol.

In this study, the ferric reducing power of plant extracts is dose-dependent as illustrated in Figure 4. The strongest activity of the reducing power is exhibited by the EtOH extract (106.23 ± 3.45 μmol Fe (II)/g DW). This is followed by WA extract (30.55 ± 2.67 μmol Fe(II)/g DW). This activity of the aqueous extracts is significantly lower than the EtOH and BHT (75.56 ± 3.57 μmol Fe (II)/g DW).

### Antibacterial activity

The antibacterial activity of the various *Urtica urens* extracts was assessed against Gram-positive (*B. subtilis*, *S. epidermidis*, *S. aureus*, *M. luteus* and *E. faecalis*) and Gram-negative (*E. coli*, *P. aeruginosa and S. enteritidis*) bacteria. The evaluation of the antibacterial activity was realized by the determination of MIC and MBC values. Table 3 reveal that all extracts vary the degrees of antibacterial activity against most of the tested Gram*-*positive and Gram-negative bacteria, and the EtOH extract was found to be the most effective. In fact, it displayed a large antimicrobial spectrum and exerted a major antibacterial effect against both tested Gram-positive and Gram-negative bacteria. While the most vulnerable bacteria for the EtOH extract were for Gram-positive (*Bacillus subtilis*, *Staphylococcus aureus*, *Micrococcus luteus* and *Staphylococcus epidermidis*), and two Gram-negative bacteria (*Salmonella enteritidis* and *Pseudomonas aeruginosa)* at concentration of 150 μg/mL. However, the WA extract did not show any effect on almost all the tested bacteria.

**Table 3.** Rapport MBC/MIC.

		Ethanol		Aqueous
	Stains	MBC/MIC		MBC/MIC
Gram+	*Bacillus subtilis*	2	bactericidal	–
	*Staphylococcus aureus*	2	bactericidal	–
	*Micrococcus luteus*	2.01	bactericidal	–
	*Staphylococcus epidermidis*	2	bactericidal	–
	*Enterococcus faecalis*	–	–	–
Gram−	*Escherichia coli*	–	–	–
	*Salmonella enteritidis*	1.93	bactericidal	–
	*Pseudomonas aeruginosa*	2	bactericidal	–
Rapport MBC/MIC Canillac & Mourey 2001		MBC/MIC ≥4		bacteriostatic
		MBC/MIC <4		bactericidal

### Vitamins D, E and C

In our results (Table 4), we noted that the concentrations of vitamin D, vitamin C and E were 1.45 ± 0.14 mg/100 g; 238 ± 2.95 mg/100 g; 356 ± 0.15 mg/100 g, respectively, in EtOH extract of *Urtica urens* and of water extract 0.23 ± 0.04; 160.55 ± 3.09; 2.3 ± 0.01 mg/100 g, respectively.

**Table 4.** Levels of vitamins D, C and E in *Urtica urens* leaves.

Sample	Vitamin D (mg/100 g)	Vitamin C (mg/100 g)	Vitamin E (mg/100 g)
Ethanol	1.45 ± 0.14	238 ± 2.95	356 ± 0.15
Aqueous	0.23 ± 0.04	160.55 ± 3.09^α***^	2.3 ± 0.01^α***^

Values are expressed as mean ± SEM of three independent determinations. ***p < 0.001.

α: compared to Ethanol.

## Discussion

The total polyphenol, flavonoid and tannin content in the EtOH and WA extracts were determined. It is well known that phenolic and flavonoid compounds contribute directly to the biological activity of plant materials (Rice-Evans et al. 1996).

With 4.768% of yield in the EtOH extract, the results of the phytochemical analysis exhibited the quantitative values of *Urtica urens* 31.41 ± 0.31 mg GAE/g E to phenolic compound, 6.81 ± 1.72 mg R/g E to flavonoids and 8.29 ± 0.3 mg GAE/g E to hydrolyzable tannin. These results demonstrate that the *Urtica urens* has high quantities of phenolic compounds. Other studies performing quantification of the phenolic compounds and flavonoids in EtOH extract of leaves showed their presence in large quantity as compared with our results for the phenolic compounds and flavonoids (Manu Kumar et al. 2013).

The content of phenolic compounds found in plants may vary during processing steps such as growing, harvesting, storage and technological procedures used (Lombardo et al. 2010). The phenolic compounds present in plants have received great attention because of their antioxidant properties and they can potentially interact with biological systems and play an important role in anticancer, anti-inflammatory, and antimicrobial activity (Wang et al. 2003; Abu-Reidah et al. 2013). The antioxidant properties are attributed to flavonoids due to their hydroxyl groups that can act as free radical scavengers, reducing agents and metal chelation (Agati et al. 2012). It has been reported that free radical-scavenging activity is greatly influenced by the phenolic composition of the sample (Cheung et al. 2003). The antioxidant activity of the WA and EtOH extracts may be attributed to their phenolic and flavonoid contents.

The antiradical activity assay is based on the reduction of 1,1-diphenyl-2-picrylhydrazyl (DPPH). The ability of the extracts and standard ascorbic acid to scavenge free radicals and pair off the odd electron was shown in this assay. It was observed that WA and EtOH extracts of *Urtica urens* are a similar scavenger of DPPH radical compared with the standard BHT. The highest inhibition effects of WA and EtOH extracts of *Urtica urens* were 64.56% and 93.56%, respectively, compared with ascorbic acid with 99.83% and BHT with 95.56% at concentration of 40 mg/L. However, in our results, further increase in the concentration of the *Urtica urens* extracts displayed increasing inhibition of DPPH radical.

According to Manu Kumar et al. (2013), using DPPH to measure the results of radical scavenging activities, *Urtica urens* leaf extract by methanol showed higher activity (78% at 0.5 mg/mL) among the other solvents and ethyl acetate displayed lower activity compared to ethanol, water and acetone.

As is well known, the antioxidant activity of plant extracts containing polyphenol components is assigned to their capacity to be donors of hydrogen atoms or electrons and to capture the free radicals (Shon et al. 2003). The DPPH radical-scavenging assay is a widely used method to evaluate the ability of plant extracts to scavenge free radicals generated from DPPH reagent (Chung et al. 2006).

Like DPPH^•^, the method of ABTS^•+^ or TEAC (Trolox Equivalent Antioxidant Capacity) is widely used to evaluate the antioxidant capacity of a variety of substances including plant extracts.

The TEAC value takes into account the capacity of a substance to react with ABTS^•+^ radical (Arts et al. 2004). The water extract caused a greater attenuation of ABTS^•+^ than DPPH^•^. This could be attributed to the fact that the ABTS^•+^ assay is aqueous based, favouring hydrophilic compounds; whereas the DPPH^•^ assay is an organic based assay, favouring hydrophobic phytoconstituents (Schlesier et al. 2002).

On the other hand, EtOH extract was ineffective regarding cellular-ROS neutralization. Instead, it caused a significant overproduction of intracellular ROS. It has been shown that *Urtica urens* can have an antioxidant or pro-oxidant effect depending on their concentration and/or structural properties.

The antioxidant activity increased with increasing extract concentration. The EtOH extract of *Urtica urens* displayed a significant (*p* < 0.001) cell-free (IC_50 _=_ _30.88 ± 3.03 μg/mL of ABTS^•+^) as comparison with WA extract (Table 2). This could be ascribed to patuletin, an abundant and potent aglycon flavonol extracted from *Urtica urens* (Ataa et al. 1995). This phytochemical is lipophilic and readily crosses the cell membrane, making it easily available in the cytosol to exert its protective antioxidant action (Abdel-Wahhab et al. 2005).

The β-carotene bleaching method is based on the loss of the orange colour of β-carotene due to its reaction with radicals formed by linoleic acid oxidation in an emulsion. The rate of β-carotene bleaching can be slowed down in the presence of antioxidants (Kulisic et al. 2004). Interestingly, the WA and EtOH extracts exhibited strong inhibition of β-carotene bleaching comparable to BHT and ascorbic acid. The control, without addition of sample, oxidized rapidly and the absorbance at 470 nm tends to zero. Therefore, the higher antioxidant activity of compounds from the *Urtica urens* extracts in this assay suggests a possible biological functionality in preventing the oxidative degradation of membrane lipids.

In this study, we used FRAP assay because it is quick and simple to perform, and its reaction is reproducible and linearly related to the molar concentration of the antioxidant(s) present. This method was initially developed to assay plasma antioxidant capacity, but could be used to measure the antioxidant capacity from a wide range of biological samples and pure compounds to fruits, wines, and animal tissues. In our study, the EtOH extract had higher ferrous reducing antioxidant power compared to standard BHT which had 75.56 ± 3.57 μmol Fe (II)/g DW (Figure 4). FRAP assay demonstrated that EtOH extract had higher activity (106.23 ± 3.45 μmol Fe (II)/g DW) than that of BHT. Based on this background, this study concludes that *Urtica urens* is considered as a potential source of natural antioxidants. The presence of general phytochemicals and specific active compounds might be responsible for their therapeutic effects. Their reducing power is probably attributed to the presence of polyphenols that may act in a similar way as reductones by donating the electrons and reacting with free radicals to convert them into more stable products and terminate the free radical chain reaction (Siddhuraju & Becker 2007). Reductones also react with certain precursors of peroxide, thus preventing the formation of the latter (Matsushige et al. 1996). The antioxidant activity has confirmed the medicinal importance of plants as naturally-occurring antioxidants (Venkatachalam & Muthukrishnan 2012).

According to Manu Kumar et al. (2013), FRAP assay exhibited ethanol fraction activity of 80 μmol Fe(II)/g DW. The antibacterial activity of *Urtica urens* extracts was evaluated against Gram-positive (*B. subtilis*, *S. epidermidis*, *S. aureus*, *M. luteus* and *E. faecalis*) and Gram-negative (*E. coli*, *P. aeruginosa* and *S. enteritidis*) bacteria. These strains are available in the laboratory. The antibacterial activity was assessed by the determination of MIC and the minimal bactericidal concentration (MBC) (the minimum concentration that inhibits all visible growth of microorganisms for 48 h at 37 °C) (Diao et al. 2014). As can be seen in Table 3, *Urtica urens* showed varying degrees of antibacterial activity against most of the Gram-positive and Gram-negative bacteria tested. The aqueous extract was found to be not active against all tested bacteria. Ethanol extract exhibited antibacterial activity (bactericidal) against Gram-positive (*Bacillus subtilis*, *Staphylococcus aureus*, *Micrococcus luteus* and *Staphylococcus epidermidis*), and two Gram-negative bacteria (*Salmonella enteritidis* and *Pseudomonas aeruginosa)* at concentration of 150 μg/mL.

The reason for the difference in sensitivity between Gram-positive and Gram-negative bacteria might be ascribed to the differences in morphological constitutions between these microorganisms; Gram-negative bacteria having an outer phospholipidic membrane carrying the structural lipopolysaccharide components. This makes the cell wall impermeable to antimicrobial chemical substances. On the other hand, Gram-positive bacteria are more susceptible, having only an outer peptidoglycan layer which is not an effective permeability barrier. Therefore, the cell walls of Gram-negative organisms are more complex in lay out than Gram-positive ones acting as a diffusional barrier and making them less susceptible to the antimicrobial agents than Gram-positive bacteria (Kaushik et al. 2015).

The most susceptible bacteria for the EtOH extract were *S. aureus, M. luteus, S. enteritidis, S. epidermidis, P. aeruginosa* and *B. subtilis* with a rapport MBC/MIC values of 2, 2.01, 1.93, 2 and 2, respectively.

These results are of great importance, particularly in the case of *S. aureus* which is well-known for being resistant to a number of antibiotics and for the production of several types of enterotoxins that cause many enteritis types and septicemia (Kaushik et al. 2015). *P. aeruginosa* is a naturally resistant Gram-negative bacteria that causes various infections in humans, and is rather insusceptible to herbal extracts (Kukric et al. 2012). Interestingly, EtOH extract showed antibacterial activity against these bacteria.

These results are in agreement with the report of Steenkamp et al. (2004) who confirmed that the aqueous extract have no activity against *Staphylococcus aureus*, *Streptococcus pyogenes*, *Escherichia coli* and *Pseudomonas aeruginosa* at concentration of 100 μg/mL. However, they reported no inhibitory activity. Moreover, according to Kaushik et al. (2015) WA extract of *Urtica urens* did not show any activity against the three tested microorganisms (*Staphylococcus aureus*, *Candida albicans*, *Escherichia coli*).

The antibacterial activity of EtOH extract of *Urtica urens* were apparently related to its terpenes and flavonoids type components, respectively, since phytochemical analysis revealed that these extracts had violet and yellow-orange spots indicating possible presence of triterpenes and flavonoids, respectively. These results support the fact that *Urtica urens*, investigated in this study, also displayed antibacterial activity.

Valuable plant source of vitamins is common nettle which is used in traditional and officinal medicine as haemostatic and vitamin substances (Dar et al. 2013). The nettle leaves are utilized mostly for the preparation of infusions. Nettle leaves contain ascorbic acid (270 mg/100 g), carotenoids (50 mg/100 g), vitamins B, D (200 mg/100 g) and E.

It was established that leaves and aqueous extract of *Urtica dioica* contain 36.402 ± 0.017 mg/100 g and 0.032 ± 0.001 mg/100 g of vitamin C, respectively (Tatyana & Valentina 2013). According to Daovy (2009), *Urtica* is a crucial source of vitamin C (20 to 60 mg/100 g), vitamins B1, B2, B5, B9, E and D. From our results, *Urtica urens* extracts are a substantial source of vitamins C, E and D.

## Conclusion

The results of this study concluded that the leaves of *Urtica urens* contain appreciable number of flavonoids, polyphenols and tannins. The extracts seem to present a real interest and potential through their antioxidant activities that have been made by the different tests (DPPH, ABTS, β-carotene and FRAP).

Their antioxidant activities further lend credence to the biological value of this plant. The EtOH extract shows activity against bacteria. This may be due to high phenolic content and the presence of active compounds such as tannins.
